# Anticoagulation in Lower Risk Atrial Fibrillation: A two Edged Sword

**DOI:** 10.5812/cardiovascmed.7380

**Published:** 2013-02-24

**Authors:** Reza Kiani

**Affiliations:** 1 Cardiovascular Intervention Research Center, Rajaie Cardiovascular Medical and Research Center, Tehran University of Medical Sciences, Tehran, IR Iran

**Keywords:** Atrial Fibrillation, Anticoagulant Drugs, Antiplatelet Drugs

*Dear Editor,*

Inside the previous issue of "Research in Cardiovascular Medicine", Arya et-al presented a nice review of novel anticoagulation strategies in patients with non-valvular atrial fibrillation (1). Through this recent review and the upcoming guidelines from various medical societies, it is plausible that the decades-long reign of coumadin in primary and secondary prevention of systemic thromboembolism in atrial fibrillation (AF) will soon come to an end. However, what is less clear out of these recent developments might be this question: How should I treat my AF patient with a lower risk of embolic events?

Lower risk AF patients may be defined as those with a CHADS2 score < 2 and they comprise the majority of the AF population (2). Although the absolute risk of embolic events is relatively low, a significant proportion of AF-related strokes occur among this group, given the high number of these patients. Traditionally, aspirin has been used for thromboprophylaxis in these patients. However, as the authors mentioned in their paper, aspirin - as an alternative to anticoagulation- may be neither a safe nor an effective option. Dual anti-platelet therapy, although more effective than aspirin alone, was inferior to warfarin in terms of both safety and efficacy. So, in lower risk AF patients with CHADS2 score < 2, the increased bleeding risk imposed by anti-platelet therapy may outweigh the risk of thromboembolism.

Warfarin therapy has been associated with a significantly decreased rate of embolic events in lower risk AF population with a relative risk reduction of at least 60%. However, high bleeding rates probably has a negative impact on the outcome of these patients. In a recent study by Poli et al. (3), risk of major bleeding in a low risk AF population on warfarin was 1.6% per year which is equal to (or even greater than) the risk of embolic events without warfarin as estimated by CHADS2 scoring system. A major drawback of warfarin is the inability to maintain the desired anticoagulation level for a long period. In a large survey in the United States for example , at least 30% of patients did not reach a stable therapeutic level as defined as time in therapeutic range > 60% (4). Hence, it is logical to assume that part of these unstable levels results in warfarin toxicity and will be associated with further increase in the rate of bleeding complications.

The introduction of novel oral anticoagulant (OAC) agents in the recent years has been associated with dramatic changes in treatment planning of non-valvular AF. However, the issue of optimal anticoagulation in lower risk AF remained unsolved. For instance, In Re-LY study the risk of major bleeding in the low to intermediate-risk subgroup on Dabigatran was approximately 3% per year while the rate of stroke was less than 1%. Also, in ARISTOTLE, nearly the same results have been reported for the lower risk subgroup on Apixaban. The ROCKET AF study for evaluation of Rivaroxaban enrolled a higher risk population with a mean CHADS2 score of 3.5, but the trial did not show a net clinical benefit over warfarin either for efficacy or safety. In a recent meta-analysis of all the three new OACs, there was no clear benefit over warfarin regarding bleeding risk reduction (5) .However; the novel agents had a remarkable impact on intra-cranial hemorrhage prevention with a risk reduction of 46% in comparison to warfarin (5). More randomized trials may be needed for a detailed evaluation of all aspects of therapy with the novel OACs.

In the last decade, various left atrial appendage (LAA) occlusion devices have been tested with acceptable procedural safety (6). This approach has a comparable efficacy to warfarin and much higher safety profile in the long run as it obviates the need for an anticoagulation regimen. Currently, these devices are reserved for intermediate or high-risk non-valvular AF especially those with a contra-indication for anticoagulation. But improvements in device technology and higher learning curve of operators may extend the applications of LAA occluders. So, in the near future there might be a role for percutaneous LAA occlusion in patients with low to intermediate risk of embolic events who have a higher chance of bleeding. Until these developments take place, individualized risk stratification should be the basis for any decision regarding the initiation of anticoagulants in this common but challenging category of patients. Combining other risk scores such as CHA2DS2-Vasc and HAS-BLED may be helpful to predict the risks and benefits of therapy more clearly.
